# The Role of NRF2 in Obesity-Associated Cardiovascular Risk Factors

**DOI:** 10.3390/antiox11020235

**Published:** 2022-01-26

**Authors:** Jorge Gutiérrez-Cuevas, Marina Galicia-Moreno, Hugo Christian Monroy-Ramírez, Ana Sandoval-Rodriguez, Jesús García-Bañuelos, Arturo Santos, Juan Armendariz-Borunda

**Affiliations:** 1 Department of Molecular Biology and Genomics, Institute for Molecular Biology in Medicine and Gene Therapy, CUCS, University of Guadalajara, Guadalajara 44340, JAL, Mexico; marina.galicia@academicos.udg.mx (M.G.-M.); hugo.monroyram@academicos.udg.mx (H.C.M.-R.); anasol44@hotmail.com (A.S.-R.); bgarcia@cucs.udg.mx (J.G.-B.); 2 Tecnologico de Monterrey, School of Medicine and Health Sciences, Campus Guadalajara, Zapopan 45201, JAL, Mexico; arturo.santos@tec.mx

**Keywords:** NRF2, obesity, cardiovascular risk factors, inflammation, oxidative stress, cardiac dysfunction, therapeutic strategies

## Abstract

The raising prevalence of obesity is associated with an increased risk for cardiovascular diseases (CVDs), particularly coronary artery disease (CAD), and heart failure, including atrial fibrillation, ventricular arrhythmias and sudden death. Obesity contributes directly to incident cardiovascular risk factors, including hyperglycemia or diabetes, dyslipidemia, and hypertension, which are involved in atherosclerosis, including structural and functional cardiac alterations, which lead to cardiac dysfunction. CVDs are the main cause of morbidity and mortality worldwide. In obesity, visceral and epicardial adipose tissue generate inflammatory cytokines and reactive oxygen species (ROS), which induce oxidative stress and contribute to the pathogenesis of CVDs. Nuclear factor erythroid 2-related factor 2 (NRF2; encoded by *Nfe2l2* gene) protects against oxidative stress and electrophilic stress. NRF2 participates in the regulation of cell inflammatory responses and lipid metabolism, including the expression of over 1000 genes in the cell under normal and stressed environments. NRF2 is downregulated in diabetes, hypertension, and inflammation. *Nfe2l2* knockout mice develop structural and functional cardiac alterations, and NRF2 deficiency in macrophages increases atherosclerosis. Given the endothelial and cardiac protective effects of NRF2 in experimental models, its activation using pharmacological or natural products is a promising therapeutic approach for obesity and CVDs. This review provides a comprehensive summary of the current knowledge on the role of NRF2 in obesity-associated cardiovascular risk factors.

## 1. Introduction

Obesity is a public health problem worldwide with constant prevalence. Obesity is an independent risk factor for cardiovascular disease (CVD), and increases the risk of dyslipidemia, insulin resistance, hypertension, and atherosclerosis. Other risk factors induced by obesity include inflammation, oxidative stress, endothelial dysfunction, heart attack, stroke, and diabetes (reviewed in [1,2]). In obesity with insulin resistance or diabetes, the heart tissue is exposed to high levels of fatty acids and carbohydrates, which are deposited in the form of lipid vesicles in the myocardium. In addition, insulin-resistance-induced hyperglycemia triggers oxidative stress and causes inflammation, thus inducing cell damage (as recently summarized by [2]). As recently reviewed by Gutierrez-Cuevas et al., obesity has been related to the development of several alterations in the myocardial structure and cardiac arrhythmias, including cardiac fibrosis, left ventricular hypertrophy (LVH), and diastolic dysfunction (reviewed in [1,2]). 

The pathogenesis of obesity and CVDs are accompanied by inflammation and oxidative stress. Body mass index (BMI) is positively correlated with oxidative stress biomarkers. Several antioxidant enzymes such as Cu–Zn superoxide dismutase (SOD1) and glutathione peroxidase 1 (GPX1) are decreased in the erythrocytes of obese patients. The increased reactive oxygen species (ROS) production is closely linked to the dysregulation of adipokines expression in adipose tissues, thus contributing to cellular dysfunction and insulin resistance (reviewed in [3,4]). Additionally, BMI may induce elevation in blood pressure (BP) by increasing cardiac output and activity of renin angiotensin aldosterone system (RAAS) [5]. As summarized by Manna et al. in hypertension induced by angiotensin II (ANG II), the production of endothelial superoxide anions is elevated due to an increase in NADPH oxidase 1 (NOX1) activity [3]. Moreover, ROS can be generated by NOX1, xanthine oxidase, and the mitochondrial oxidative phosphorylation system in the adipose tissue, and both pro-inflammatory cytokine and ROS contribute to progression of vascular damage (reviewed in [3,4]). In fact, hyperglycemia-induced ROS accumulation and hyperglycemia cause endothelial cell apoptosis and inflammation, resulting in endothelial injury (reviewed in [6]). The pathogenesis of atherosclerosis includes oxidative stress, high levels of oxidized lipoproteins, immune reactivity, and inflammation, as well (reviewed in [3,7,8]). Importantly, vascular endothelial dysfunction caused by dyslipidemia is considered the main factor for the beginning of atherosclerosis (reviewed in [8,9]). 

Nuclear factor erythroid 2-related factor (NRF2; encoded by *Nfe2l2* gene), and with the approved name NFE2-like bZIP transcription factor 2 by the HUGO Gene Nomenclature Committee (HGNC), is a key transcription factor of redox regulation. In this review, we use the official symbol *Nfe2l2* gene to refer to NRF2. NRF2 is regulated by kelch-like ECH associated protein 1 (KEAP1) and has a short half-life of approximately 10–30 min in normal conditions [10,11,12]. Under oxidative stress, KEAP1 is detached from its complex with NRF2. Therefore, NRF2 is accumulated in the nucleus and binds to a DNA sequence called antioxidant response element (ARE), thus activating the upregulation of cytoprotective antioxidative genes, including NAD(P)H quinone dehydrogenase 1 (*Nqo1*), *Hmox1*, which expresses the hemoxygenase-1 (HO-1) protein, and previously known as heat shock protein 32 (HSP32), glutathione S-transferases (*Gsts*), catalase (*Cat*), *Gpx1*, and *Sod1*, among others (reviewed in [13,14,15]). As reviewed by Matzinger et al. in obesity models using *Nfe2l2*-knockout (*Nfe2l2*-KO) mice, ROS levels and blood glucose levels augment, and insulin signaling is impaired [16]. Cardiac *Nfe2l2* expression is significantly downregulated in diabetic animals and patients (reviewed in [17]). NRF2 has a protective role against oxidative injury of the myocardium. *Nfe2l2*-KO mice develop cardiac hypertrophy, LV diastolic dysfunction, and impaired calcium homeostasis [18]. *Nfe2l2*-KO also results in accelerated heart failure following ischemic injury, suggesting that NRF2 deficiency could be involved in the early onset of heart failure in humans (reviewed in [19,20]). As summarized by Mikhailov and Torrado, NRF2 expression is overregulated during early stages of cardiac hypertrophy but decreased at mild pathological hypertrophy [20]. In models of hypertrophic cardiomyopathy with forced expression of NRF2 and its target genes, left ventricular (LV) remodeling and fibrosis are significantly reversed. Similarly, NRF2 overexpression by phytochemical intake decreases diastolic dysfunction in hypertensive Dahl salt-sensitive rats with heart failure and preserved ejection fraction (HFpEF) (reviewed in [20]). Geraniol is another phytochemical that activates NRF2 and has protective effects on oxidative, inflammatory, and apoptotic alterations in isoproterenol-induced myocardial infarction (reviewed in [15]). The pharmacological activation of NRF2 using the synthetic triterpenoid CDDO-Im (2-Cyano-3,12-dioxooleana-1,9-dien-28-imidazolide) results in the prevention of high fat diet (HFD)-induced obesity in mice models, as reviewed in detail by Mimura and Itoh [21]. As reviewed by Chen and Maltagliati, other NRF2 inducers such as sulforaphane are capable of suppressing atherosclerotic signaling, while natural compounds with effects in NRF2 activation such as resveratrol or curcumin have a protective effect against HFD-induced oxidative stress, apoptosis, inflammation, and endothelial cell dysfunction [19].

Here, we aim to cover the usual functions of NRF2 on adipogenesis and obesity, including obesity-associated cardiovascular risk factors. This review also discusses the current state of NRF2 as a therapeutic strategy for the prevention and treatment of CVDs.

## 2. Structural Features and Properties of NRF2

NRF2 was first discovered in 1994 during research on β-globin gene regulation (reviewed in [22,23]). The human *Nfe2l2* gene is localized in the chromosome 2q31.2 region. The commonly studied NRF2 protein comprises 605 amino acids, which are translated from an mRNA 3352 nucleotide (nt) long containing 1055 nt as 5′-untranslated region (5′-UTR) and 479 nt in the 3′-UTR. In addition, the *Nfe2l2* sequence is conserved between species. Compared to human *Nfe2l2*, there is homology of 99.9 with chimpanzee, 91.4 with cat, 89.1 with cattle, 88.8 with dog sequence, 83.2 with rat, 82.5 with mouse, and 49.1% with zebrafish (reviewed in [14,19]). In 1991, a discrete regulatory element was discovered in the promoter region of others genes encoding phase II drug metabolism enzymes. This cis-regulatory element was named the antioxidant response element (ARE). In 1997, the consensus ARE sequence was elucidated as 5′-TMAnnRTGAYnnnGCR-3′, where M = A or C; R = A or G or T; Y = C or T; n = any. This consensus element was subsequently described in several promoters of genes encoding phase II drug metabolism enzymes, including cytoprotective genes (reviewed in [19,24]). As reviewed by Zang et al., the transcription of *Nfe2l2* may be activated by itself and other transcription factors such as the aryl hydrocarbon receptor (AHR), peroxisome proliferator-activated receptor alpha (PPARA) or gamma (PPARG), nuclear factor-κB (NF-κB), specificity protein 1 (SP-1), p53, myocyte-specific enhancer factor 2 D (MEF2D), c-JUN, c-MYC, and breast cancer 1 (BRCA1) [25]. In addition, epigenetic regulations are involved in *Nfe2l2* transcriptional control, such as methylation in CpG islands of the *Nfe2l2* promoter or H3 histone, and acetylation of H4 histone (reviewed in [25]). Interestingly, as reviewed in detail by Chen and Maltagliati, several functional *Nfe2l2* polymorphisms are associated with risk of human cardiovascular disorders, for example, rs6721961 is a C-to-A transversion at −650 nt location and results in diminished ARE activity in the *Nfe2l2* gene promoter [19]. This polymorphism was studied in a Finnish cohort study and was found associated with a higher risk of cerebrovascular disease and hypertension. In Japan, it was found that cardiovascular mortality in -686A/A carriers is higher than G/G or G/A carriers, and this promoter polymorphism (rs35652124) was consistent with decreased NRF2 expression (reviewed in [19]). Several polymorphisms have been characterized in the intron regions. For instance, individuals with G/G homozygotes have lower triglyceride levels and reduced risk of cardiovascular mortality compared with individuals who are G/C heterozygous or C/C homozygous at rs2364723 (reviewed in [19]). However, the mechanism involved in the contribution of these observed phenotypes remains unclear.

**Figure 1:**
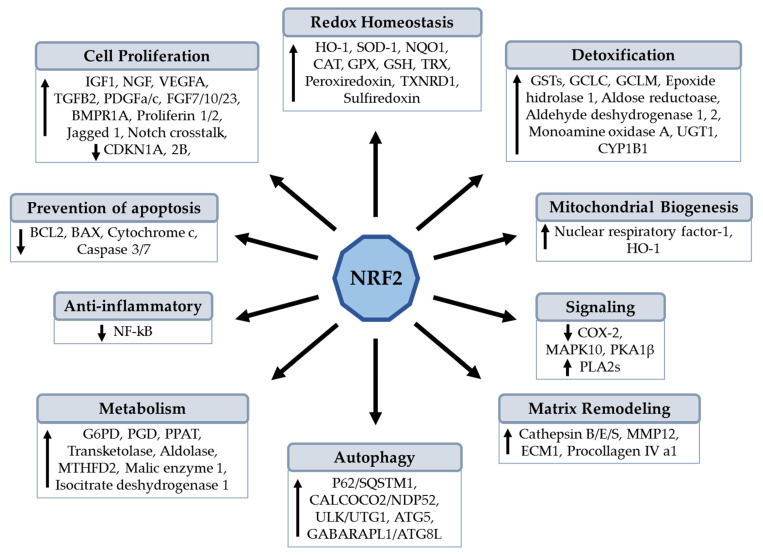
NRF2 protein regulates different cellular functions based on the genes under its control. ATG5, autophagy related 5; BAX, BCL2 associated X, apoptosis regulator; BCL2, BCL2 apoptosis regulator; BMPR1A, bone morphogenetic protein receptor 1A; CALCOCO2/NDP52, calcium binding and coiled-coil domain 2, also called nuclear dot protein 52 kDa; CDKN1A and 2B, cyclin-dependent kinase inhibitor 1A and 2B; CYP1B1, cytochrome P450 family 1 subfamily B member 1; ECM1, extracellular matrix protein 1; FGF7/10/23, fibroblast growth factor 7, 10, and 23; G6PD, glucose-6-phosphate dehydrogenase; GABARAPL1/ATG8L, GABA type A receptor associated protein like 1, also called ATG8-like; GSTs, glutathione S-transferases; IGF1, insulin-like growth factor 1; MAPK10, mitogen-activated protein kinase 10; MMP12, matrix metalloproteinase 12; UGT1A, UDP glucuronosyltransferase family 1 member A complex locus; MTHFD2, methylenetetrahydrofolate dehydrogenase (NADP+ dependent) 2, methenyltetrahydrofolate cyclohydrolase; NGF, nerve growth factor; P62/SQSTM1, p62 protein, also called sequestosome 1; PKA1β, protein kinase a1β; PLA2s, phospholipases A2; PPAT, phosphoribosyl pyrophosphate amidotransferase; TGFB2, transforming growth factor beta 2; ULK1/ATG1, unc-51-like autophagy activating kinase 1 and is homologous to ATG1 in yeast; VEGF, vascular endothelial growth factor A.

Genetic animal models have been designed to study the loss- and gain-of-function to provide clues in the understanding of the role and impact of NRF2 on the structure and function of heart tissue. NRF2 is a master transcription factor which is expressed in most of the tissues, including the cardiovascular system, where it is an essential endogenous suppressor of oxidative stress in both cardiomyocytes and cardiac fibroblasts. NRF2/ARE signaling is highly conserved in all species and regulates the expression of over 1000 genes in the cell under normal and stressed conditions, including cytoprotective and detoxifying phase II enzymes (reviewed in [20,22,23,25]). NRF2 also protects cellular components such as proteins and DNA from oxidative insults. NRF2 regulates the proteasomal degradation of proteins, endoplasmic reticulum (ER) stress, cell proliferation, and metabolic reprogramming. Other functions showed by NRF2 include autophagy, anti-inflammatory activity, and inflammasome signaling, as well as apoptosis, mitochondrial biogenesis, hematopoiesis, metabolism, and stem cell survival (reviewed in detail and reported by [19,23,24,25,26,27,28,29,30,31,32,33,34,35,36,37]); see Figure 1.

### 2.1. NRF2 Overexpression in Heart Tissue

Redox homeostasis regulates various cellular functions in both physiological and pathological conditions. NRF2 has been considered a critical regulator of cellular defense against various pathological insults in the heart. However, several studies have shown a detrimental role of NRF2 in cardiac disease progression (reviewed in [25]). Cardiac-specific overexpression of *Nfe2l2* (ca*Nfe2l2*) in mouse models have been used to induce pro-reductive and reductive stress, and these mice exhibited increased expression of genes such as glutamate-cysteine ligase catalytic subunit (*Gclc*), glutamate-cysteine ligase modifier subunit (*Gclm*), glutathione-disulfide reductase (*Gsr*) with previous name glutathione reductase, *Nqo1*, GST Mu 1 (*Gstm1*), and *Gpx1*, including the glutathione (*Gsh*), which is a key regulator of the cellular thiol redox state [38]. Furthermore, constitutive NRF2 activity increased the expression of CAT, GPX3, monoamine oxidase A (MAOA), thioredoxin reductase 1 (TXNRD1), thioredoxin 1 (TRX1), peroxiredoxin 6 (PRDX6), and phosphogluconate dehydrogenase (PGD). Additionally, ca*Nfe2l2* mice showed increased expression of the hypertrophic genes natriuretic peptides A and B (*Nppa*, *Nppb*), among other genes [39]. The chronic reductive stress triggered by ca*Nfe2l2* probably leads to pathological cardiac remodeling [38,39]. However, as recently reviewed by Zhou et al., another study showed that cardiomyocyte restricted transgenic overexpression of NRF2 protects against myocardial oxidative stress, cell death, fibrosis, hypertrophy, and dysfunction in mice with pressure overload induced by 4 weeks of transverse aortic arch constriction (reviewed in [4]).

### 2.2. Inhibition of NRF2 Expression in Heart Tissue

**Figure 2:**
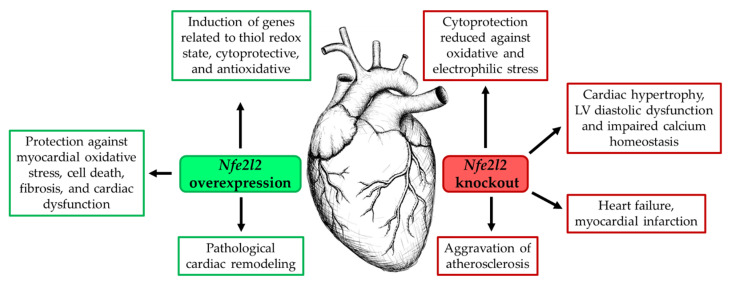
Effects of NRF2 overexpression and NRF2 deficiency on cardiac tissue. Cardiac-specific transgenic expression of *Nfe2l2* induces antioxidative genes and protection against cardiac dysfunction. However, constitutive expression of *Nfe2l2* leads to the development of a hyper-reductive state, which can result in pathological cardiac remodeling. NRF2 deficiency causes oxidative and electrophilic stress, including cardiac dysfunction and heart failure. Only confirmed effects are shown.

The disruption of the *Nfe2l2* gene does not cause any apparent structural or functional abnormalities in neonatal and early postnatal heart under non-stressed physiological conditions (reviewed in [20]). However, as reviewed by Ma, several studies have clearly shown that *Nfe2l2*-KO mice substantially increase susceptibility to exogenous oxidative challenges and disease conditions associated with oxidative pathology [24]. Furthermore, the ablation of both *Nfe2l1* and *Nfe2l2* result in embryonic lethality because of severe oxidative stress and extensive apoptosis [40]. In line, as reviewed by Zang et al. *Nfe2l2*-KO neonatal mouse ventricular cardiomyocytes do not provided cytoprotection against oxidative and electrophilic stress [25]. Although the myocardium of young Nfe2l2-KO mice retains the expression of antioxidant genes (*Cat*, *Nqo1*, *Hmox1*, *Gpx1*, *Gsr*, *Gclm*, and *Gclc*), the mice exhibited poor compensatory response to acute exercise stress and develop atrial hypertrophy in response to high-intensity exercise stress due to impaired cellular antioxidant defenses [41,42]. Moreover, cardiomyocytes from Nfe2l2-KO mice treated with high glucose concentrations exhibited greater ROS production and apoptosis compared with cardiomyocytes from *Nfe2l2* wild type (WT) animals (reviewed in [25]). As reviewed by Mikhailov and Torrado, other findings reported in NRF2-deficient models include a rapid onset of cardiac dysfunction during experimental pressure overload (associated to transverse aortic constriction) or regional ischemic injury (associated to cardiac artery occlusion) in young adult mice (2-month-old). These results indicate that NRF2 inhibition can increase the susceptibility of heart tissue to oxidative stress and thus lead to cardiac dysfunction [20]. In addition, the NRF2 signaling pathway has been found downregulated in aging, diabetes, hypertension, and chronic inflammation, which are risk factors for CVDs (reviewed in [19]). Studies in *Nfe2l2*-KO mice demonstrated that *Nfe2l2* gene deficiency leads to LV diastolic dysfunction with mild cardiac hypertrophy but preserved systolic function and heart failure following myocardial infarction. NRF2 deficiency in humans appears to be linked to the early onset of heart failure [20,43]. Additionally, as reviewed by Chen and Maltagliati, the lack of NRF2 in macrophages aggravates atherosclerosis, including an increased sensitivity to cardiac hypertrophy by ANG II or pressure overload [19], as seen in Figure 2. Therefore, NRF2 is a critical regulator of the defense response against ROS and an important transcriptional factor for myocardial cytoprotection.

## 3. Role of NRF2 in Obesity-Induced Cardiac Alterations and Adipogenesis

The development of CVD is intimately linked to the pathogenesis of obesity, which includes an increase in the levels of circulating free fatty acid (FFA; including palmitate), ROS and reactive nitrogen species (RNS), causing chronic inflammation, which is another important source of oxidative stress, and insulin resistance [16,18]. Regarding oxidative stress, BMI is positively associated to indicators of protein, lipid, and DNA oxidative damage (advanced oxidation protein products (AOPP), malondialdehyde (MDA) or 8-iso-prostaglandin F2α (8-iso-PGF2α), and 8-hydroxy-2′-deoxyguanosine (8-OHdG) during obesity (reviewed in [44]). Moreover, epicardial fat is a source of ROS and inflammatory status, which are closely linked to obesity-related cardiac dysfunction [45,46]. Of note, obesity is an important risk factor for cardiac fibrosis, including structural and functional cardiac alterations, and heart failure, which increase the risk of sudden death. As recently reviewed by Gutierrez-Cuevas et al., the increased cardiac work as a consequence of obesity elevates the blood volume, stroke volume, and cardiac output, which may result in LV dilatation and LVH, causing the development of coronary heart disease (CHD) and atrial fibrillation, heart failure, and ischemic stroke [2]. Furthermore, oxidative stress from epicardial fat may play a role in the genesis of atrial fibrillation (reviewed in [46]). Obesity also induces LV remodeling, leading to both systolic and, particularly, diastolic dysfunctions. Additionally, obesity increases the risk of heart failure due to the enlargement of the left atrium, caused by both increased blood volume and diastolic ventricular dysfunction (reviewed in [2]). Other alterations induced by obesity in the myocardial structure include LV remodeling with increased wall thickness and mass, including increased thickness and volume of the right ventricle (reviewed in [2]).

Obesity is also an independent risk factor for HFpEF (reviewed in [47]). Epidemiological studies have reported that HFpEF accounts for approximately half of all heart failure cases. Cardiac hypertrophy is characterized by myocardial fibrosis (excess deposition of extracellular matrix: ECM), capillary rarefaction, inflammation, and cellular dysfunction, which results in maladaptive ventricular remodeling (accumulation of cardiac fibroblasts) and heart failure. Maladaptive hypertrophy is induced by a sustained vascular hypertension, involving ANG II [48]. In addition, oxidative stress has emerged as a key mechanistic mediator of myocardial fibrosis [49]. In line, as reviewed by Faria et al., the activation of NF-κB and ROS are involved in cardiac cell apoptosis, fibrosis, and hypertrophy in high serum glucose condition or obesity, leading to severe cardiac malfunction and the development of heart failure [50]. p65, an NF-κB subunit, inhibits the transcription of *Nfe2l2* and its target genes [51]. The transforming growth factor beta 1 (TGFB1) is upregulated in the hearts of HFD-fed rats with a decreased activity of NRF2 signaling. Therefore, the persistent production of ROS leads to NRF2 downregulation, which stimulates the overexpression of TGFB1, thus aggravating the myocardial fibrosis [52]. In fact, the activation of TGFB1 pathway itself can inhibit the NRF2-mediated expression of genes encoding phase II detoxifying proteins, causing more production of ROS [53]. Importantly, the overactivation of ROS accelerates cardiac fibrosis along with cardiac dysfunction; thus, inhibiting ROS production could partially prevent cardiac dysfunction [54]. With respect to this, the overexpression of *Nfe2l2* inhibited cardiac fibrosis and dysfunction; meanwhile, *Nfe2l2*-KO mice develop cardiac fibrosis and dysfunction after transverse aortic constriction surgery [55]. Additionally, LV diastolic dysfunction in *Nfe2l2*-KO mice is associated with cardiac hypertrophy and the downregulation of the sarcoplasmic reticulum Ca(2+)-ATPase (SERCA2a) in the myocardium [18]. In addition, NRF2 deficiency increased ANG II-induced oxidative stress [56]. Therefore, both effects together induce cardiac injury (maladaptation and dysfunction). As reviewed by Mikhailov and Torrado, NRF2 expression is increased during early stages of cardiac hypertrophy but decreased at mild pathological hypertrophy (commonly associated with HFpEF) [20]. In addition, in several models of hypertrophic cardiomyopathy, the overexpression of NRF2, including its target genes (*Hmox1*, *Gpx1*, *Txnrd1*, *Nqo1*, and *Sod2*: mitochondrial), significantly reverses LV remodeling and fibrosis (reviewed by [20]). These results indicate that NRF2 is an important factor in preventing the heart from oxidative stress-related damage, and NRF2 activation might reverse cardiac fibrosis and cardiac dysfunction. However, the molecular mechanisms involved between cardiac remodeling and NRF2 remains unclear.

Adipose tissue dysfunction contributes to the development of CVD risk factors and obesity-associated CVD pathogenesis. NRF2 is abundantly expressed in white adipocyte tissue (WAT) [57]. *Nfe2l2*-KO mice showed decreased adipose tissue with small adipocytes and are protected against weight gain and obesity induced by HFD [58]. In addition, the selective deficiency of NRF2 impairs adipocyte differentiation in mouse embryonic fibroblasts, 3T3-L1 cells, and human subcutaneous preadipocytes, decreasing the expression of PPARG, CCAAT enhancer binding protein alpha (CEBPA), and their downstream targets. On the contrary, the transfection of *Nfe2l2* stimulates *Pparg* promoter activity, and the knockdown of *Keap1* (*Keap1*-KD; activation of NRF2) enhances PPARG expression in 3T3-L1 cells, which accelerates hormone-induced adipocyte differentiation [58]. In agreement, adipocyte differentiation was impaired in *Nfe2l2*-KD 3T3-L1 cells, whereas *Nfe2l2*-KD in 3T3-L1 cells blocked the stimulated adipogenesis generated by the deficiency of KEAP1. In addition, *Nfe2l2*-KD led to the decreased expression of CCAAT enhancer binding protein β (CEBPB). Therefore, these findings suggests that NRF2 is one of the transcription factors involved in the early events of adipogenesis by regulating the expression of CEBPB [59]. Since aryl hydrocarbon receptor (AHR) negatively controls adipocyte differentiation, it is suggested that NRF2 would inhibit adipogenesis through interaction with the AHR pathway [34]. Another study showed that *Nfe2l2*-KO mice developed a phenotype with less insulin-resistance, and *Nfe2l2*-KO mice had higher plasma levels of fibroblast growth factor 21 (FGF21) and higher FGF21 mRNA levels in liver and WAT compared to WT mice [60]. FGF21 is considered a hormone that regulates energy metabolism, glucose tolerance and adipose tissue expansion. These results were later confirmed when *Nfe2l2*-KO mice were fed an HFD for 14 weeks, after which mice exhibited reduced blood glucose, decreased number but increased size of adipocytes, accompanied with elevated expression of many proteins related with an increase in glucose, lipid, and energy metabolism in the visceral fat-like FGF21 and PPARG coactivator 1 alpha (PPARGC1A; also known as PGC1a). An elevated adipocyte maturation (delta-like 1 homolog: DLK1) and a reduced adipogenesis (PPARG, CEBPA) were also showed [61]. In another study conducted on HFD-induced obese mice, it was showed that *Nfe2l2*-disrupted mice gained less weight than WT mice [62]. Interestingly, mice with adipocytes or hepatocytes cell-specific deletion of *Nfe2l2* were fed an HFD for 6 months showed similar increases in body weight and body fat content. However, the deletion of *Nfe2l2* in adipocytes led to a worsened systemic metabolic phenotype (increased glucose, cholesterol, and non-esterified fatty acids levels), whereas the deletion of *Nfe2l2* in hepatocytes modestly reduced insulinemia after long-term HFD-induced obesity in mice [63]. Adipocyte-specific *Nfe2l2*-KO mice with a leptin-deficient ob/ob background (a model with an extremely positive energy balance) led to reduced WAT mass but with severe metabolic syndrome and serious insulin resistance, hyperglycemia, and hypertriglyceridemia [64]. Interestingly, rifampicin treatment in adult male mice fed an HFD developed insulin resistance but reduced fat accumulation and decreased expression of several adipogenic genes in WAT. Furthermore, in 3T3-L1 preadipocytes, rifampicin decreased the expression of *Cebpβ*, *Pparg*, and *Cebpa* genes in the early and/or later stage of hormonal-cocktail-induced adipogenesis. Mechanistically, rifampicin inhibits *Nfe2l2*-ARE luciferase reporter activity and expression of NRF2 downstream genes in the early stage of adipogenesis in 3T3-L1 preadipocytes [65]. It was reported that oxidative stress is markedly increased in WAT from mice with HFD-induced or genetically (ob/ob)-induced obesity and from human individuals with obesity. Furthermore, NRF2 expression and activity were induced in response to oxidative stress, recruiting NRF2 to the *Srebf1* (sterol regulatory element binding transcription factor 1) promoter, which induces target gene transcription and subsequent lipogenesis, thus promoting lipid accumulation in adipocytes and exacerbating the development of obesity. In contrast, *Nfe2l2* ablation ameliorated oxidative stress-induced lipid accumulation [66]. Therefore, these findings indicate that NRF2 deficiency protects against lipid accumulation in adipose tissue.

Studies of NRF2 functions related to lipid metabolism in adipose tissue have been reported. For instance, constitutive NRF2 activation in leptin-deficient mice (with *Keap1*-KD) exhibited less lipid accumulation and reduced lipogenic gene expression. Meanwhile, constitutive NRF2 activation inhibited lipid accumulation in WAT and adipogenesis induced insulin resistance in Lep(ob/ob) mice. Moreover, pharmacological NRF2 activation by sulforaphane prevented adipogenesis and lipid accumulation [67]. In line, treatment with the synthetic CDDO-Im effectively prevented HFD-induced increases in body weight, adipose mass, and hepatic lipid accumulation in WT mice but not in *Nfe2l2*-disrupted mice [62]. Subsequently, other natural and synthetic NRF2 activators have been proven effective against obesity. For example, oltipraz, a potent NRF2 activator, can prevent body weight and fat gain induced by an HFD. In addition, parthenolide and epigallocatechin 3-gallate showed the capacity to inhibit obesity and obesity-induced inflammatory responses via NRF2/KEAP1 signaling under HFD conditions [68,69]. In line, treatment with CDDO-methyl ester (CDDOMe), another NRF2 activator, decreases total body fat, plasma lipids levels, FFA levels and improves glucose tolerance and insulin resistance in HFD-fed mice. CDDO-Me activates protein kinase AMP-activated catalytic subunit alpha 2 (PRKAA2); also known as AMPK via liver kinase B1 (LKB1; also known as serine/threonine kinase 11, STK11) induction both in vivo and in vitro [70]. Finally, brassinin, another NRF2 activator, inhibits lipid accumulation at the late stage of preadipocytes differentiation through the inhibition of *Cebpa* and PPARG. Furthermore, in a co-culture model of 3T3-L1 cells and RAW264.7 cells, brassinin-induced NRF2 activation increases the levels of adiponectin, HO-1, interleukin 6 (IL6), and C-C motif chemokine ligand 2 (CCL2; also known as monocyte chemoattractant protein-1: MCP-1) and improves obesity-induced inflammation in both adipocytes and macrophages [71], thus indicating a protective function of NRF2 activation against obesity. In contradiction, it was reported that when fed an HFD (HFD; 10% lard, 2% cholesterol, 0.5% bile salt, and 87.5% base forage) for 8 weeks, *Nfe2l2*-KO mice gained slightly more weight than the WT mice without statistical difference [72]. Another study investigated the effect on the NRF2 pathway of HFD-induced (39.7 kcal% fat) obesity among *Nfe2l2*-KO, WT, and *Keap1*-KD mice and concluded that the genetic alteration of *Nfe2l2* does not prevent diet-induced obesity in mice [73]. Hormone fluctuations might be considered important in the development of obesity. For instance, in an ovariectomized menopausal model using *Nfe2l2*-KO mice, it was found that *Nfe2l2* deletion and a decline in estrogen levels promoted an increase in bodyweight, which may be related with increased glucose and decreased serotonin (5-HT) levels [74]. Therefore, it is possible that diet composition (especially fat content), differences in cell passage number, experimental design, mice genetic background and feeding period may be responsible for the conflicting results related to the role of NRF2 in adipogenesis and obesity. NRF2 effects on obesity are summarized in the Table 1.

## 4. NRF2 and Cardiovascular Risk Factors

The major effects of obesity on cardiovascular health are mediated through risk factors such as insulin-resistance, hyperglycemia, dyslipidemia, and hypertension. An absence of these risk factors in obese individuals may not be associated with increased mortality risk (reviewed in [75]). Oxidative stress plays a critical role as a factor linking obesity with its associated complications, and the possible contributors to oxidative stress in obesity include hyperglycemia, elevated tissue lipid levels, vitamin and mineral deficiencies, hyperleptinemia, chronic inflammation, endothelial dysfunction, and impaired mitochondrial function, as reviewed in detail by Manna and Jain [3].

### 4.1. NRF2 and Hyperglycemia

The excessive consumption of fat and carbohydrates in food induces a prolonged oxidative stress and inflammation in obese people. Obesity is intimately linked to hyperglycemia and insulin resistance. It is well-know that most ROS are originated from mitochondria when there is incomplete reduction of oxygen during oxidative phosphorylation. In addition, glucose metabolism can generate ROS through the involvement of several pathways: (1) the formation of advanced glycation end-products (AGEs), (2) the activation of protein kinase-C (PKC), (3) glyceraldehyde auto-oxidation, (4) increased polyol pathway activity, (5) increased hexosamina metabolism, and 6) the increased production of ANG II (reviewed in [76]). In agreement, treatment of H9c2 cells with high glucose increased ROS level and caspase-3 activity [77]. In addition, high glucose treatment in human coronary artery endothelial cells inhibited the NRF2/ARE signal pathway, which led to ROS accumulation [78]. The production of ROS due to high levels of glucose in cardiomyocytes may contribute to the development of cardiomyopathy. According to these data, NRF2 has a critical role against high glucose-induced oxidative damage in cardiomyocytes by inducing cytoprotective genes such as *Nqo1* and *Hmox1* [79]. Interestingly, high glucose can stimulate NRF2 protein expression and translocation into the nucleus in cardiomyocytes through PKCα/PKCδ-ROS-JNK/p38 signaling; thus acting as defense against oxidative stress [80]. Moreover, diallyl trisulfide which is a powerful antioxidant among the sulfur-containing compounds of garlic oil, increased NRF2 protein stability and nuclear translocation, protecting against hyperglycemia-induced ROS-mediated apoptosis by upregulating the PI3K/AKT1/NRF2 pathway in cardiomyocytes exposed to high glucose [77]. Another study showed that aldose reductase inhibition by fidarestat, increased the high glucose-induced expression of antioxidant enzymes such as SOD1 and CAT and the activation of PRKAA1 or also named AMPK-α1 in Thp1 cells. Therefore, the pretreatment with aldose reductase inhibitor prepares the monocytes against hyperglycemic stress by overexpressing the NRF2-dependent antioxidative proteins [81].

On the other hand, Takeda G protein-receptor-5 (TGR5; also known as GPBA, MBAR, or GPBAR1) is activated by bile acids and has effects on the regulation of glucose metabolism, energy balance, inflammation, digestion, and sensation. In addition, bile acids increase energy expenditure and prevent the development of obesity and insulin resistance, which are mediated by TGR5 [82]. The activation of TGR5 protects against high glucose-induced cardiomyocyte injury by inhibiting inflammation and apoptosis, due to TGR5 suppresses the NF-κB pathway and activates the NRF2 pathway [83].

*Nfe2l2* deficiency in Lep(ob/ob) mice has been reported to reduce insulin-stimulated AKT1 phosphorylation along with a slight decrease in glucose transporter type 4 (GLUT4) expression, which reduced WAT mass and prevented hepatic lipid accumulation but induced insulin resistance and dyslipidemia [84]. However, adipocyte-specific *Nfe2l2*-KO mice fed with high fat food did not show insulin resistance [85]. In accordance with this, NRF2 deficiency improves glucose tolerance in mice fed with an HFD, whereas NRF2 activation worsens glucose tolerance in mice fed either a control diet or an HFD [73]. Furthermore, sirtuin 1 (SIRT1) produced by visceral WAT is a critical factor in preserving improved glucose tolerance in NRF2 deficiency mice during obesity [86]. Interestingly, we reported in male C57BL/6 J mice with obesity induced by high-fat/high-carbohydrate diet that prolonged-release pirfenidone decreased *Nfe2l2* gene expression, and these mice showed improved systemic insulin sensitivity [87].

During insulin biosynthesis within β cells due to hyperglycemia, the formation of three disulfide bonds per one insulin molecule results in the release of millions of ROS molecules per minute; consequently, damage caused by ROS can contribute to β cell dysfunction. In addition, β cells from early-stage type 2 diabetic rats exhibited increased NRF2 levels, suggesting that the NRF2 pathway is activated to respond to high ROS levels (reviewed in [76]). We also reported that NRF2 is overexpressed in cardiac tissue from mice obesity induced by high-fat/high-carbohydrate diet for 16 weeks [87]. Interestingly, β cell-specific deletion of *Keap1* (increased NRF2 levels) in inducible nitric oxide synthase (iNOS)-transgenic mice have restored insulin secretion from pancreatic β-cells in the context of reactive species damage, thus NRF2 positively regulates β cell function [76]. Vitexin (apigenin-8-C-glucoside) is a flavone c-glycoside, present in many edible and medicinal plants, that protects β-cells against high glucose toxicity by improving insulin secretion and by activating key proteins such as NF-κB and NRF2, thus regulating apoptosis [88]. Therefore, several findings suggest that NRF2 has an important role in the regulation of glucose metabolism.

### 4.2. NRF2 and Hyperlipidemia

Several studies have shown that excessive circulating free fatty acids (FFAs), such as palmitate, induce ROS production via protein kinase C (PKC)-dependent activation of NOX1. In smooth muscle cells and endothelial cells, this leads to increased oxidative stress and decreased mitochondrial oxidative capacity, which may cause morphological and functional changes in tissues (reviewed in [4,82]). In human umbilical vein endothelial cells (HUVECs), treatment with cyanidin 3-O-glucoside, which is a natural anthocyanin, inhibited the NF-κB pro-inflammatory pathway and adhesion molecules, including improving intracellular redox status induced by palmitate, and these effects were attributed to the activation of the NRF2/electrophile responsive element (EpRE) pathway [89]. Furthermore, other medicinal plants have been shown to reduce hyperlipidemia. For instance, Herba houttuyniae was reported to have effects on decreasing serum lipids and tissue damage markers, ameliorated hepatic lipid accumulation, improved cardiac remodeling, and decreased hepatic and cardiac oxidative stress induced by hyperlipidemia in male C57BL/6 J mice. Furthermore, NRF2 and PPARG were downregulated in hyperlipidemic mouse livers and hearts, which may be attributable to the loss of PRKAA2 activity. All of these changes were reversed by Herba houttuyniae supplementation [90]. Curcumin increases HO-1 via activation of NRF2, inducing to attenuation of HFD-induced elevations of malonedialdeyde (MDA) and ROS in skeletal muscle but not in adipose tissue or liver [91,92]. As reviewed by Zhang et al., NRF2 can attenuate hyperlipidemia by activating the PRKAA2 signaling pathway [93]. The treatment of Pediococcus pentococcus PP04 reduced the increase in total cholesterol, triglycerides, low-density lipoprotein cholesterol (LDL-cholesterol), FFAs, and leptin in C57BL/6N mice fed with an HFD. Pediococcus pentococcus PP04 improved HFD-induced hyperlipidemia and oxidative stress by triggering PRKAA2 and the NRF2/CYP2E1 signaling pathway [94].

### 4.3. Role of NRF2 in Endothelial Dysfunction

ROS generation in the cardiovascular system helps to maintain endothelial and vascular smooth muscle cell (VSMC) functions; vascular tone control; inflammation-related responses; cell growth and proliferation, including modulation of extracellular matrix production; apoptosis; and angiogenesis (reviewed in [95,96]. In vascular cells, ROS generation can be activated by several stimuli such as cytokines, ANG II, endothelin 1 (EDN1), aldosterone, and platelet-derived growth factor (PDGF) [97]. Furthermore, ROS alter the balance between endothelial proliferation and apoptosis, which can cause excessive angiogenesis or the loss of endothelial cells (reviewed in [98]), thereby contributing the pathophysiology of the response to injury.

It is important to note that vascular endothelium is a main source for several oxidant enzymes, which include NOX1, xanthine oxidase, and NO synthase (reviewed in [3]). Obesity and hyperglycemia increase ROS accumulation, and insulin resistance. High glucose levels increase NOX1 enzyme activity and endothelial nitric oxide synthase (eNOS) uncoupling, thus contributing to high ROS levels and impaired vascular function [99,100,101]. In addition to ROS accumulation, high glucose also mediates cell viability reduction and NRF2/ARE signal pathway inhibition via the upregulation of KEAP1 expression and the downregulation of monomethyltransferase SET8 expression in HUVECs. Meanwhile, SET8 overexpression ameliorated high-glucose-induced KEAP1/NRF2/ARE pathway inhibition and endothelial oxidation [102].

Additionally, as recently reviewed by Abu-Saleh et al., HUVECs exposed to high glucose and ox-LDL exhibited the activation of the EDN1 system and attenuated eNOS and NRF2 level/activity [103]. However, another study reported that HUVECs exposed to high glucose showed increased levels of ROS and upregulated NOX2, NOX4, NRF2, and NQO1 effects that were reversed by the PPARB/D agonists GW0742 and L165041 [104]. In the same study, it was shown that PPARB/D activation induced vascular protection against hyperglycemia-induced oxidative stress by suppressing NOX2 and NOX4 expression, including a direct induction of HO-1 and the subsequent downregulation of the NRF2 pathway [104]. In cardiac tissue with steatosis from a mouse NASH model induced by a high-fat/high-carbohydrate diet, we found elevated NRF2 protein levels, and the treatment with prolonged-release pirfenidone overexpressed PPARA and PPARG proteins, while NRF2 protein was decreased, thereby improving myocardial steatosis, hypertrophy, and fibrosis induced by obesity [87]. In support of previous mentioned studies, hyperglycemia-mediated oxidative stress activates NRF2 and its target genes in coronary arterial endothelial cells, and HFD-induced vascular ROS and endothelial dysfunction are even more intense in *Nfe2l2*-KO mice, indicating that the activation of NRF2 pathways confers endothelial protection under obesity and diabetes conditions (reviewed in [16,105]). In line with this, as reviewed by Gupte et al., *Nfe2l2*-KD impairs the proliferation, adhesion, and migration of cultured human coronary arterial endothelial cells, indicating that NRF2 is essential for normal angiogenic endothelial processes [105]. The NRF2 activator magnesium lithospermate B suppresses glucose-induced proliferation and migration of aortic VSMCs and prevents neointimal hyperplasia after catheter-induced arterial injury in diabetic rats, thereby suggesting vascular protective effects (reviewed in [105]). Many NRF2 activators have been reported, for instance, the activation of NRF2 by sulforaphane prevented biochemical dysfunction and related functional responses of endothelial cells induced by hyperglycemia in which transketolase expression was increased [106]. Cinnamaldehyde is a key flavor compound in cinnamon essential oil and protects from endothelial dysfunction under high glucose conditions and its effect is mediated by NRF2 activation [107]. Baicalin is the main component found in Scutellaria baicalensis root, an herb widely used in traditional Chinese medicine, which can alleviate hyperglycemia-induced endothelial impairment by downregulating ROS and inflammation via the AKT1/GSK3B/FYN-mediated NRF2 activation [108]. Caffeic acid, a dietary hydroxycinnamic acid abundant in coffee, has been reported to upregulate NRF2/EpRE and prevent endothelial dysfunction associated with inflammation and oxidative stress induced by high concentration of glucose [109].

### 4.4. NRF2 and Atherosclerosis

Atherosclerosis is a chronic inflammatory disease of large or medium-sized muscular arteries, and contains an arterial plaque mainly made up of fat, cholesterol and calcium hardens, inflammatory cells, and smooth muscle cells (SMCs) and their secreted products, such as collagen and elastin, thus narrowing the vessel lumen and obstructing the flow of oxygen-rich blood (reviewed in [16,21]). It is developed due to chronic injury and activation of endothelial cells through different insults such as hyperglycemia, high serum LDL-cholesterol, low HDL-cholesterol, hypertension, hyperlipidemia, smoking, oxidative stress, and inflammation. Atherosclerosis is the main cause for myocardial infarction, stroke, and perivascular artery disease and is accelerated by diabetes (reviewed in [16]).

On the other hand, circulating miRNAs have been reported deregulated in severe obesity. For example, morbidly obese patients showed a significant increase in miR-140-5p, miR142-3p, and miR-222 and decreased levels of miR-532–5p, miR-125b, miR130b, miR-221, miR-15a, miR-423-5p, and miR520c-3p [110]. Interestingly, miR-140-5p aggravated hypertension and oxidative stress of mice with atherosclerosis via targeting NRF2 and SIRT2. Meanwhile, the downregulation of miR-140-5p reduced oxidative stress and ROS levels by activating the protein expression of NRF2, SIRT2, KEAP1, and HO-1 in vitro [111]. The biomarkers for atherosclerosis include 8-OHdG (marker of oxidative DNA damage), MDA (marker of lipid peroxides), carboxymethyl lysine and glutarin (markers of glycosylation), nitrotyrosine (markers of nitric oxide), SOD1, GPX1, CAT, HO-1, thioxygenin, and oxyphosphatase. Among the proteins with antioxidant activity regulated by NRF2 are SOD1, CAT, HO-1, GPX1, and NQO1 (reviewed in [112,113]). Elevated ROS levels leads the oxidation of LDL to ox-LDL that contributes to oxidative stress and foam cell formation in the arterial wall, reviewed in detail by Mimura and Itoh [20]. NRF2 is considered a protective transcriptional factor since it induces the expression of many antioxidant genes that may ameliorate atherosclerosis progression. Supporting the atheroprotective role of NRF2, it is activated by laminar shear stress, thus exerting anti-atherogenic effects (reviewed in [21]). *Gpx1*-deficient mice, which is a NRF2 target gene, exhibited an increased ox-LDL-induced foam cell formation, indicating that this gene is atheroprotective [114]. However, male *Nfe2l2*-deficiency in *ApoE*-KO mice, a model of atherosclerosis not associated with obesity, showed less atherosclerotic lesions only at late stages; this finding could be related to regulatory effects of NRF-2 on lipid metabolic genes [115,116,117]. Intriguingly, in *ApoE*-KO mice with *Hmox1* deletion, the atherosclerosis and oxidative stress were accelerated [118]. Additionally, oxidized lipids induce NRF2-dependent CD36 scavenger receptor expression in macrophages, which results in the intracellular accumulation of ox-LDL [119]. However, the absence of NRF2 in HFD myeloid-derived macrophages [120] or the deletion of *Nfe2l2* in myeloid cells of *LDL* receptor KO mice [121] aggravates atherosclerotic lesions and increases pro-inflammatory genes expression, indicating that NRF2 modulates the pro-inflammatory vascular milieu associated with atherosclerosis. NRF2 anti-atherogenic effects have been related to its modulatory properties on migration and proliferation of VSMCs [122]. NRF2 depletion enhanced VSMCs migration in response to PDGF, and *Nfe2l2*-deficient mice showed higher neointimal hyperplasia in a wire injury model [122]. Furthermore, the NRF2/KEAP1 system modulates VSMC apoptosis during neointimal formation and, consequently, inhibits neointimal hyperplasia after vascular injury [123]. Moreover, NRF2 target genes reduce VSMCs proliferation [124,125], and NRF2 activity is important to maintain VSMCs phenotype [126]. Importantly, the activation of the KEAP-1/NRF2/NQO1 pathway decreases VSMCs circulating calciprotein particles-induced VSMCs calcification [127]. In summary, these findings show that NRF2 functions as an anti-atherogenic factor by reducing VSMCs migration, proliferation, calcification, and vascular remodeling. In addition, NRF2 induces anti-oxidative genes and inhibits inflammatory genes in response to various signals, including laminar shear stress (reviewed in [21,112]).

Several natural compounds and molecules have been shown to activate NRF2 and provide a promising new option to prevent and treat atherosclerosis. Natural flavone acacetin is an anti-inflammatory, antiarrhythmic, and is commonly used in the treatment of myocarditis. *ApoE*-KO mice fed with Western diet and treated with acacetin showed protection against atherosclerosis through NRF2 pathway, which increased reductase levels in circulation and aortic roots, decreased plasma inflammatory factor levels, as well as accelerated lipid metabolism [128]. Berberine in *ApoE*-KO mice inhibits atherosclerotic disease development due to the repression of macrophage foam cell formation through suppressing AP-1 activity and activation of NRF2/HO-1 pathway [129]. Lunasin, a soybean-derived 43-aa peptide, upregulates HO-1 via the PI3K/AKT1/NRF2/ARE pathway and reduces H2O2-induced ROS production in vascular endothelial cells (VECs), thereby attenuating oxidant-induced endothelial injury and inhibiting atherosclerotic plaque progression in *ApoE*-KO mice [130]. FGF21 attenuates inflammation and oxidative stress in atherosclerotic rats via the NRF2-ARE signaling pathway [131].

### 4.5. NRF2 in Hypertension

Under pathophysiological conditions of obesity, the renin–angiotensin–aldosterone system (RAAS) is activated and amplifies inflammation and structural remodeling, thereby inducing cardiac and vascular damage, and other structural alterations which results in cardiac dysfunction, recently reviewed in details by Gutierrez-Cuevas et al. [1]. The RAAS has an important role in cardiovascular system hemostasis through the regulation of blood pressure (BP), vasoconstriction, sodium intake, and potassium excretion. Inappropriate activation of the RAAS has been related with a profound hypertension and cardiovascular morbidity (reviewed and reported in [1,132]). For instance, hyperglycemia and impaired insulin regulation increase ANG II, causing to myocardial hypertrophy, fibrosis, and apoptosis. In addition, ANG II elevates the expression of NOX1, which is associated with endothelial dysfunction, and vascular remodeling. NOX1 is a significant source of ROS in cardiovascular diseases, including ANG II-dependent hypertension [1,23,132]. The activation of NOXs by ANG II involves protein kinase C (PKC) and c-SRC-dependent pathways, which result in ROS formation (reviewed in [133]). The oligopeptide Leu-Ser-Gly-Tyr-Gly-Pro (LSGYGP) from tilapia was shown to protect against ANG II-induced HUVECs injury through the reduction of oxidative stress and alleviate endothelial damage. LSGYGP suppressed the expression of the NO, ROS, and NF-κB pathways and reduced protein levels such as iNOS, cyclooxygenase-2 (COX-2) and EDN1. It also decreased the expression of gamma-glutamyltransferase 1 (GGT1) and HO-1, as well as increased SOD1 and GSH expression through the NRF2 pathway [134]. SIRT6 expression was reportedly decreased in vascular endothelial cells exposed to ANG II, and SIRT6 overexpression ameliorates ANG II-induced apoptosis and oxidative stress in vascular endothelial cells by activating NRF2 antioxidant signaling. Thus, SIRT6 could be a potential therapeutic target for treating hypertension associated with endothelial dysfunction [135]. As reviewed by Barancik et al., NRF2 may be important in BP regulation by induction of HO-1 gene expression, which can lower BP in model of spontaneously hypertensive rats (SHR) [136]. HO-1 is the rate-limiting enzyme in the degradation of the heme complex, which generates carbon monoxide (CO), biliverdin, and iron. CO regulates vascular response, inhibiting the vasoconstrictor, EDN1, and/or by inducing soluble guanylate cyclase and increasing cGMP (with vasodilatory effects) [137]. Biliverdin may be transformed to bilirubin (a potent antioxidant), and both can directly inhibit NOX1 activity, thus contributing to the actions of HO-1 to sustain vascular homeostasis [138].

Several studies have shown correlative findings between NRF2 and ROS in vessels and heart in hypertension animal models (reviewed in [139]). NRF2 and NRF2-regulated antioxidant enzymes were found downregulated in an SHR model of hypertension, which increased oxidative stress and vascular dysfunction (reviewed in [17,22]). These results could be related to BACH1 expression, an NRF2 corepressor, which is increased when ANG II is stimulated [140]. Interestingly, there were no differences in basal blood pressure between *Nfe2l2*-KO and WT mice, including ANG II-induced BP elevation in *Nfe2l2*-KO mice compared with WT mice [141]. However, sulforaphane, an NRF2 activator, improves the redox imbalance observed in the vasculature of SHR, and diet with a sulforaphane precursor decreased vascular oxidative stress, improving endothelial-dependent vasodilatation and lowering BP (reviewed in [17]). Another NRF2 activator, resveratrol, decreased oxidative stress and attenuated the severity and hypertension progression in SHR (reviewed in [17]). Other NRF2 activators have been reported to have beneficial effects on hypertension. Nitrite decreases BP and improves vascular dysfunction in hypertension by activating the NRF2 pathway, which increased the mRNA expression of NRF2-regulated genes including *Sod1*, *Cat, Gpx1*, *Trx1*, and *Trx2* [142]. Nitrite’s effects on antioxidant responses in hypertension could be explicable at least in part by the downregulation of NOXs and vascular xanthine oxidoreductase (XOR) [143,144,145]. Interestingly, hydrogen sulfide (H2S) improves endothelial dysfunction by reducing the activation of NLRP3 inflammasome and oxidative stress in SHR, and after knocking down *Nfe2l2*, the protective effect of H2S was eliminated [146].

Diosmetin is a citrus flavonoid with antioxidant and anti-inflammatory effects. Diosmetin improved hypertension, endothelial dysfunction by modulation of NRF2/HO-1, and p-JNK/p-NF-κB expression in hypertensive rats [147]. Irisin is an exercise-induced myokine, and it was reported that intravenous injection of irisin in an SHR model reduced BP, plasma norepinephrine, paraventricular nucleus (PVN) levels of neuronal activation, oxidative stress, and inflammation by activating the NRF2 signaling pathway in the hypothalamic paraventricular nucleus [148]. Furthermore, NRF2-mediated anti-oxidation in the PVN can decrease sympathetic activity and ameliorate hypertension [149]. On the other hand, *Arrb1*-KO mice are susceptible to diet-induced obesity, and these mice develop increased fat mass accumulation and decreased whole-body insulin sensitivity [150]. The overexpression of ARRB1 improved the cardiovascular dysfunction and decreased BP in SHR by facilitating the Nrf2 activation and by decreasing the level of ROS in the rostral ventrolateral medulla (RVLM), a key region for BP regulation [151]. Moreover, selective *Nfe2l2* gene deletion in the RVLM elevates BP, increasing sympathetic outflow and impairing baroreflex function, potentially through impaired antioxidant enzyme activities [152]. Additionally, 4-month-old male rat offspring prenatally exposed to dexamethasone and fed a high-fat diet in postnatal state showed aggravated prenatal dexamethasone-induced hypertension, which was prevented by dimethyl fumarate treatment. Dimethyl fumarato induced an increase in renal *Nfe2l2* gene expression, reduction of oxidative stress, plasma asymmetric dimethylarginine (ADMA) and renal soluble epoxide hydrolase protein levels, as well as the activation of genes linked to nutrient sensing and autophagy (*Pparb*, *Pparg*, *Pgc-1α*, *Ulk1*, and *Atg5*) [153]. In line with this, maternal NRF2 activation by dimethyl fumarate protects male adult offspring against hypertension induced by combined dexamethasone and high-fat exposures. Dimethyl fumarato reduced oxidative stress, ADMA levels, and downregulated the renin-angiotensin system (*Ren*, *Agt*, *Ace*, and *Agtr1a*), including increasing renal protein levels of certain nutrient-sensing pathways and autophagy [154]. In accordance with this, the NRF2 inhibition induces oxidative stress, renal inflammation, and hypertension by diminishing phase II antioxidant enzymes in mice [155]. In summary, NRF2 may be vasoprotective in hypertension and is important in blood pressure regulation under hypertension. Therefore, the NRF2 transcriptional factor may have a therapeutic potential for hypertension.

## 5. Role of Oxidative Stress and Inflammatory Response in CVD

ROS and RNS play a crucial role in CVD pathogenesis and in the understanding of the mechanisms that originated it. Several pieces of evidence showed that the main source of ROS is vascular wall, mainly through mitochondrial electron transport chain activity, NOX1, xanthine oxidase, and uncoupled nitric oxide synthase (NOS) (reviewed in [17]). Vascular cells produce ROS when stimulated by cytokines, ANG II, EDN1, aldosterone, and PDGF, favoring oxidative modifications of redox-sensitive proteins, such as calcium-calmodulin kinase, ryanodine receptor, and sarcoplasmic reticulum ATPase, thus altering their conformation, stability, activity, and ability to react with other proteins and modulate vascular function (reviewed in [90]). ROS generation is important to preserve endothelial and vascular smooth muscle cells function, vascular tone, responses related to inflammation process, cell growth, proliferation, apoptosis, and angiogenesis (reviewed in [112]). However, when an imbalance between ROS production and the ability to counteract the damage induced by them by antioxidants defenses, the risk to develop CVD increases (reviewed in [112]).

NRF2 is a nuclear factor that plays an important protective role against the oxidative stress generated during CVD development. As reviewed by Chen and Maltagliati, during atherosclerosis, the deposition of cholesterol, lipids, and inflammatory responses in specific areas of blood vessels promote endothelial damage and the proliferation of smooth muscle cells [19]. NRF2 activation is linked with atherosclerosis resistance [156]. Pre-clinical experiments show that a lack of NRF2 in macrophages allow a pro-atherogenic foam cell formation and leads atherosclerosis development [121]. CVDs generally occur in ancient people. Harman’s theory explain that aging is the result of impairment accumulation produced by excessive oxidative (reviewed in [157]). In addition, vascular aging is accompanied with chronic oxidative damage that results in a failure to activate ARE-induced gene expression in glutamate-cysteine ligase catalytic subunit (*Gclc*) and modulatory (*Gclm*) subunits [158]. As reviewed by Kloska et al., the relation between NRF2 and aging persists in several species, such as humans, rats, and mice [159]. In a study by Ungvari et al., the role of NRF2 in detoxification mechanisms in the vasculature of male rats of 3, 12, 18, 24, and 28 months old was studied [160]. Using culture arteries isolated from young and old rats the authors observed that in aorta of Fischer 344 Brown Norway rats, aging results in a progressive increase in O_2_^.-^ production. In addition, the downregulation of NRF2 protein and mRNA, which involves a decrease in gene expression of *Nqo1*, γ-glutamylcysteine synthase, and *Hmox1*. On the other hand, another nuclear factor affected by aging and the changes on NRF2 activity is NF-κB and its target genes such as intercellular adhesion molecule-1 (ICAM-1) and IL6 [160]. Another important mechanism associated with CVD development that involves NRF2 participation is mitochondrial dysfunction, which includes bioenergetic defects, altered mitochondrial dynamics, and impaired transcription [161]. In myocardia, this damage process often elicits disrupted energy metabolism and stimulates ROS production (reviewed in [162]). NRF2 has an important role in the mitochondrial dysfunction modulation. For example, during the dysfunction of mitochondrial permeability transition pore (MPTP), an excess of calcium flow into the mitochondrial matrix lead to a calcium overload and irreversible damage to cardiomyocytes under abnormal conditions. Sulforaphane, an upstream mediator of NRF2, increases the resistance of mitochondria to MPTP opening. This observation suggests that NRF2 has a protective role against mitochondrial dysfunction [163].

The inflammatory process negatively complements the damage caused by ROS in cardiac tissue. An erroneous NRF2 signaling pathway increases the susceptibility to several inflammatory conditions, such as atherosclerosis or other cardiac affections (reviewed in [162]). Evidence shows that lipopolysaccharide can induce the expression of iNOS in macrophages of *Nfe2l2*-KO mice, resulting in an aggravated hypertrophy of cardiomyocytes. Another mechanism is correlated with the expression of *Ccl2* and 1b (*Ccl21b*), which are diminished in macrophages from WT mice but not in *Nfe2l2*-KO mice, suggesting a relation between this signaling pathway that led to inflammation with the myocardial hypertrophy via NRF2 activation [164].

It is well known that there is a relationship between metabolic disorders and CVD. As reviewed by Rani et al., during obesity, the oxidative system is activated through NOX1 and ER stress in adipocytes, favoring a chronic inflammatory scenario [165]. Evidence showed that NF-κB and NRF2 had an important relationship in cellular inflammatory response. Oxidative stress can induce inflammatory response through the activation of transcription factors such as NF-κB [166]. Zeng et al. evaluated the role between NF-κB and NRF2, and the ability of curcumin to prevent these cardiac alterations induced by obesity, using an in vitro model in cardiac H9C2 cells exposed to palmitate and an in vivo model of HFD-fed male mice. This study shows that curcumin administration was effective to increase NRF2 expression and inhibit NF-κB activation in both experimental models. In addition, curcumin administration was effective to prevent the high expression of IkB-α in heart tissues, and mRNA expression of tumor necrosis factor (TNF; also known as TNF-α), IL1B, and IL6. Similarly, this drug has effective responses to increase NRF2 expression in the hearts of mice fed an HF diet and promote a significant increase in the mRNA and protein expression of HO-1 and NQO1 [167].

**Figure 3:**
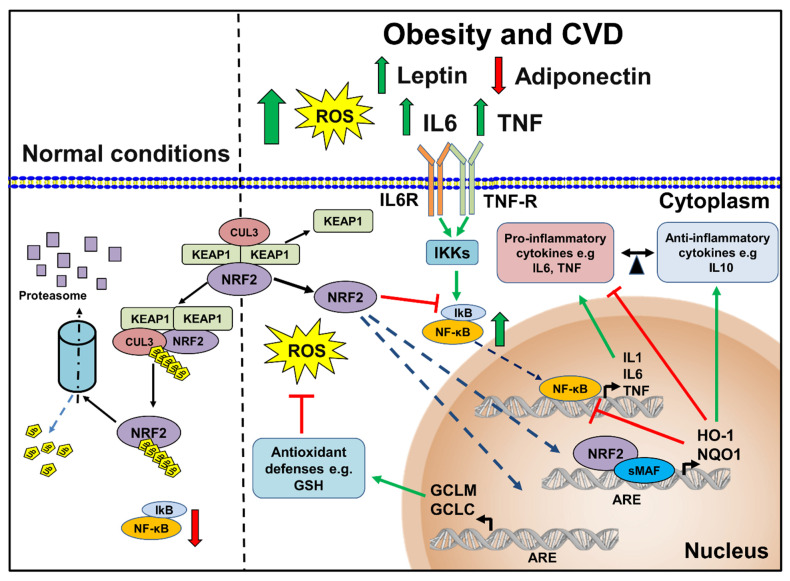
Regulation of NRF2 under normal conditions compared to the situation in obesity and CVD. In normal conditions, NRF2 remains inactively bound to its endogenous inhibitor KEAP1; later, this heterodimer will bind to CUL3-RBX E3 ubiquitin ligase complex triggering NRF2 degradation by the proteasome. Under conditions of obesity and development of CVD, including increased leptin, decreased adiponectin, further increased pro-inflammatory cytokines (IL6 and TNF), and increased ROS, NRF2 is released from KEAP1 and translocated to nucleus, forming a heterodimer with sMAF; KEAP1-sMAF complex binds to ARE sequences, promoting the expression of antioxidant (*Gclm and Gclc*) and anti-inflammatory (*Hmox1* and *Nqo1*) genes. Additionally, the activation of HO-1 and NQO1 inhibit the pro-inflammatory activity of NF-κB. ARE, antioxidant response element; CUL3, cullin 3; CVD, cardiovascular disease; Gclc, glutamate-cysteine ligase catalytic subunit; Gclm, glutamate-cysteine ligase, modifier subunit; HO-1, heme oxygenase 1; IkB, inhibitor of κB; IKKs, inhibitory kappa B kinases; IL6, interleukine 6; IL6R, interleukine 6 receptor; IL10, interleukine 10; KEAP1, kelch-like ECH associated protein 1; NF-κB, nuclear factor-κB; NRF2, nuclear factor erythroid 2-related factor 2; Nqo1, NAD(P)H quinone dehydrogenase 1; sMAF, small Maf proteins; TNF, tumor necrosis factor; TNF-R, tumor necrosis factor receptor.

As reviewed by Saha et al., NF-κB induces the expression of several pro-inflammatory cytokines (IL1, IL6, and TNF), COX-2, iNOS, and vascular adhesion proteins [168]. Interestingly, NRF2 negatively regulates NF-κB signaling through different mechanisms: first, decreasing ROS levels, NRF2 blocks NF-κB activation; second, NRF2 prevents proteasomal degradation of IkB and subsequently inhibits NF-κB nuclear translocation (reviewed in [168]). Pre-clinical studies have demonstrated that cardamonin, a flavonoid with a wide range of biological activities, was effective at preventing oxidative and inflammatory cardiotoxic damage induced by doxorubicin administration. These responses were mediated by an activation of the NRF2 signaling pathway, suppressing apoptotic cell death, and inhibiting pro-inflammatory response [169]. Some of the mechanisms are described in Figure 3.

In summary, oxidative stress and inflammatory response are two processes closely related with cardiovascular diseases. Transcription factors NRF2 and NF-κB participate in these processes; the first, trying to counteract the harmful response of the second, in this way positively modulates the damage process. These transcription factors should be considered important pharmacological targets for the development of new treatments for the benefit of patients with CVD.

## 6. Therapeutic Strategies Implicated in the NRF2 Activation

Drugs and dietary phytochemicals have been identified as potent activators of cellular antioxidant genes through the induction of the NRF2 signaling pathway, thus providing a beneficial therapeutic effect in a variety of cells and animal models. Since the NRF2 pathway modulates several genes involved in glucose and lipid metabolism, many NRF2 activators have been tested in order to uncover the therapeutic effects of NRF2 on obesity and its comorbidities. For instance, the epigallocatechin 3-gallate-induced activation of NRF2 in liver, as well as in the adipose tissue of obese mice, improves lipid levels, decreases oxidative products generation, and reduces body mass, insulin, and glucose levels [68]. To note, as reviewed by da Costa et al., the improvement of the metabolic profile is beneficial in improving cardiovascular function [112]. Another interesting NRF2 activator is curcumin, which decreases oxidative stress, inflammation, fibrosis, hypertrophy, and tissue remodeling induced by HFD. Curcumin also inhibits oxidative stress, inflammation, and hypertrophy induced by FFAs exposure of cardiac cells (reviewed in [112]). In addition, as reviewed by Vasileva et al., the pharmacological activation of NRF2 in high fat and fructose diet-consuming mice by the tricyclic cyanoenone inducer TBE-31 causes the inhibition of inflammation, reduces lipogenesis, and prevents hepatosteatosis [14]. Other NRF2 inducers have been tested for reducing infarct size, preserving LV function, and reducing adverse events such as arrhythmia in animal models of myocardial infarction. Examples of these compounds include sulforaphane, fumarate, resveratrol, alpha-lipoic acid, a synthetic curcumin analog, 4-hydroxy-2-nonenal, and prostaglandin D2. In a rat model of postinfarct arrhythmia, lithium, a GSK-3 inhibitor, was reported to activate NRF2 and protect against ventricular arrhythmias (reviewed in [19]). Table 2 shows several NRF2 activators with different beneficial effects on cardiovascular risk factors and cardiac dysfunction commonly associated with obesity. However, clinical studies are needed to evaluate the NRF2 activators considered in Table 2 in order to confirm its therapeutic effects seen in preclinical studies. With respect to this issue, bardoxolone methyl, an NRF2-activating substance, and also known as CDDO-Me, was analyzed in 2185 patients with type 2 diabetes and stage 4 chronic kidney disease. This study reported that bardoxolone methyl did not reduce the risk of the primary composite outcome of end-stage renal disease (ESRD) death from cardiovascular causes. In addition, significantly increased risks of heart failure and of the composite cardiovascular outcome (nonfatal myocardial infarction, nonfatal stroke, hospitalization for heart failure, or death from cardiovascular causes) prompted the termination of the trial (NCT01351675) [170].

## 7. Conclusions

Obesity-associated CVD developments are orchestrated by the dysregulation of adipokines and other adipose-derived signaling molecules due to adipose tissue dysfunction, increasing FFAs levels, and ROS and pro-inflammatory cytokines production, which are exacerbated by numerous pathological conditions associated with obesity, such as hyperglycemia, dyslipidemia, oxidative stress, endothelial dysfunction, hypertension, and atherosclerosis. Insulin resistance is probably one of the most important cardiovascular risk factors implicated in cardiac dysfunction. NRF2 is a key regulator of oxidative stress and is considered a master transcription factor of antioxidant defense and is thus an attractive therapeutic target for cardiovascular diseases. In addition, NRF2 signaling modulates several physiological processes associated with the maintenance of cellular homeostasis (see Figure 1). NRF2 is reportedly deregulated in adipose tissue during obesity, and this transcriptional factor modulates the progression of atherosclerosis, inflammation and lipid homeostasis, including other cardiovascular risk factors associated with obesity. Therefore, the pharmacological or phytochemical activation of NRF2 mitigates obesity, insulin resistance, and other comorbidities, including cardiac remodeling and fibrosis, as well as diastolic dysfunction, thus improving cardiac dysfunction. However, NRF2 inducers are not clinically available because of biosafety concerns, and few are in clinical development. Future work should be directed to clarify the role of NRF2 activity in the complex context of obesity, considering the presence of cardiovascular risk factors. Clinical trials are needed to find compounds with a good pharmacokinetic/pharmacodynamic profile for treatment of obesity and CVD.

## Figures and Tables

**Table 1:** Obesity models with activation or deficiency of NRF2.

Mouse and Cell Line Models	Experimental Conditons	Main Effects Reported
*Nfe2l2*-KO male mice	Obesity induced by an HFD for 12 weeks	Reduced adipose tissue mass, Impaired adipocyte differentiation [58]
*Nfe2l2*-KO male mice	Mice were fed an HFD for 180 days	Mice were partially protected from HFD-induced obesity and developed a less insulin-resistant phenotype [60]
*Nfe2l2*-KO male mice	Obesity induced by an HFD for 8 weeks	Mice showed an accelerated onset of obesity and NASH via the induction of hepatic IR. In addition, mice had increased in total and hepatic weight [72]
*Nfe2l2*-KO male mice	Obesity induced by a high-fat Western diet for 12 weeks	Mice were resistant to high-fat, Western-diet-induced glucose intolerance. Absence of NRF2 activity did not prevent diet-induced obesity [73]
*Nfe2l2*-KO mice	Obesity induced by an HFD for 12 weeks	Mice showed significant oxidative stress in the WAT. Body weight and WAT weight were significantly lower in *Nfe2l2*-KO mice, including triglycerides content in the liver and muscle [66]
Male mice with adipose-specific ablation of *Nfe2l2*	Obesity induced by an HFD for 14 weeks	Body weight and body fat content of *Nfe2l2*-KO mice showed comparable results with *Nfe2l2* control mice, but exhibited reduced blood glucose, reduced number but increased size of adipocytes [61]
*Nfe2l2*-KO ovariectomized female mice	Ovariectomy was performed once the mice reached 15-16 weeks old	*Nfe2l2*-KO ovariectomized mice had a greater body weight gain, an increase in blood glucose level, and a reduction in LDL and the level of 5-HT [74]
*Nfe2l2*-KD in primary cultured mouse preadipocytes and 3T3-L1 cells	Mouse primary preadipocytes were isolated from WAT. Lentiviral transduction of 3T3-L1 cells with particles for shRNA targeting NRF2	Hampered adipogenic differentiation induced by hormonal cocktails [59]
Male mice with cell-specific deletion of *Nfe2l2* in adipocytes (ANKO) or hepatocytes (HeNKO)	Obesity induced by an HFD for 24 weeks	Mice showed similar increases in body weight and body fat content. ANKO mice showed high fasting glucose levels and high levels of cholesterol and nonesterified fatty acids. HeNKO mice showed low insulin levels and trended toward improved insulin sensitivity without having any difference in liver triglyceride accumulation [63]
Adipocyte-specific *Nfe2l2*-knockout male and female mice on a leptin-deficient ob/ob background	Monitored during an 11-week period in mice 4–15 weeks of age	Mice showed reduced WAT mass but severe metabolic syndrome with aggravated insulin resistance, hyperglycemia, and hypertriglyceridemia [64]
*Keap1*-KD male mice, which have increased NRF2 activity	Obesity induced by a high-fat Western diet for 12 weeks	Mice exhibited prolonged elevation of circulating glucose during a glucose tolerance test. Enhancement of NRF2 activity did not prevent diet-induced obesity [73]
NRF2 overexpression or *Keap1*-KD in 3T3-L1 cells	Lentiviral transduction of 3T3-L1 cells with particles for shRNAs targeting NRF2	Accelerates hormone-induced adipocyte differentiation [58]
Lep(ob/ob)-*Keap1*-KD mice, which have increased NRF2 activity	*Keap1*-KD mice were fed an HFD for 36 days	Lep(ob/ob)-*Keap1*-KD mice exhibited less lipid accumulation, smaller adipocytes, decreased food intake, and reduced lipogenic gene expression. Obesity and lipid accumulation in white adipose tissue was decreased in *Keap1*-KD mice. Constitutive NRF2 activation inhibited lipid accumulation in WAT, suppressed adipogenesis, induced insulin resistance and glucose intolerance, and increased hepatic steatosis in Lep(ob/ob) mice [67]
Male C57BL/6 J mice	Obesity induced by an HFD for 17 weeks with or without ECGC, an NRF2 activator	Dietary EGCG significantly reduced weight gain, plasma glucose, insulin level, liver and kidney weight. Prevention of HFD-induced AGEs formation [68]
Male C57BL/6 J mice	Obesity induced by an HFD for 12 weeks with or without parthenolide, an NRF2 activator	Parthenolide-administered mice showed a significant reduction in body weightand WAT. Parthenolide inhibitedobesity-induced inflammatory responses [69]

Abbreviations: AGEs, advanced glycation end products; EGCG, epigallocatechin 3-gallate; HFD, high fat diet; 5-HT, serotonin; IR, insulin resistance; Keap1, kelch-like ECH associated protein 1; KD, knockdown; KO, knockout; LDL, low-density lipoprotein; NASH, non-alcoholic steatohepatitis; *Nfe2l2*, NFE2 like bZIP transcription factor 2; Nrf2, nuclear factor erythroid 2-related factor; WAT, white adipocyte tissue.

**Table 2:** Activators of NRF2 analyzed in cardiovascular risk factors and cardiac dysfunction.

Drug or Compound	Representative Model	Effects Reported	Pathological Condition
Acacetin	EA.hy926 cells and apolipoprotein E deficiency (*ApoE*^−/−^) female mice with Western diet	In cells, decreased ROS. In vivo, attenuated atherosclerosis by increasing reductase levels and aortic roots, decreasing plasma inflammatory factor levels	Atherosclerosis [128]
Antrodin C	HUVECs	Prevented high glucose-induced senescence, ameliorated ROS and apoptosis	Hyperglycemia [171]
Asiatic acid	Cultured cardiac fibroblasts. Male WKY rats and male SHRs	In vitro, inhibited ANG II-induced cardiac fibrosis. In vivo, attenuated myocardial hypertrophy, reduced collage deposition, MDA, and ROS	Hypertension [172]
Brassinin	3T3-L1 and RAW264.7 cells	Suppressed lipid accumulation, decreased inflammatory cytokines and ROS	Obesity [71]
Chalcone L6H9	H9C2 cells and male C57BL/6 mice with STZ-induced diabetes	In vitro, reduced inflammation, ROS, mitochondrial dysfunction, cell apoptosis, fibrosis, and hypertrophy. In vivo, decreased cardiac cytokines and ROS level, decreasing cardiac apoptosis, hypertrophy, and fibrosis	Hyperglycemia [173]
Chrysin	Male Sprague Dawley rats	Attenuated myocardial oxidative stress via upregulating eNOS and NRF2 target genes	Obesity [174]
Curcumin	H9C2 cells and male C57BL/6 mice	In vitro, decreased ROS, inflammation, apoptosis, and hypertrophy. In vivo, suppressed oxidative stress, inflammation, apoptosis, fibrosis, hypertrophy, and tissue remodeling	Obesity [167]
Cyanidin-3-O-glucosid	HUVECs	Via NRF2/BACH1 and NF-κB pathways, improved intracellular redox status, inhibited NF-κB proinflammatory pathway and adhesion molecules	Endothelial dysfunction induced by palmitic acid [89]
Equol	HUVECs and apolipoprotein E knockout (*ApoE*^−/−^) male mice fed a HFD	In cells, inhibited apoptosis induced by t-BHP and thapsigargin, attenuated ER stress markers. In vivo, reduced triglycerides, total cholesterol, and LDL-cholesterol and increased HDL-cholesterol	Atherosclerosis [175]
Herba houttuyniae	Male C57BL/6 J mice	Via activation of the PRKAA2/PPARG/NRF2 cascade, attenuated lipids, improved cardiac remodeling, and ameliorated cardiac oxidative stress	Hyperlipidemia [90]
Imidazopyridine derivative X22	H9c2 cells and male Wistar rats	Inhibited ROS, inflammation, apoptosis, fibrosis, and hypertrophy. NF-κB also was inhibited	Obesity [176]
Irisin	Cardiac fibroblasts and male C57BL/6 mice	Attenuated ANG II-induced cardiac fibrosis via NRF2 mediated inhibition of ROS/TGFB1/SMAD2/3 signaling axis	Cardiac dysfunction [177]
JC-5411 (Phenethyl isothiocyanate formulation)	Apolipoprotein E deficient (*ApoE*^−/−^) male mice	Reduced atherosclerotic plaque area in both in face aorta and aortic sinus through suppression of inflammation and regulation of lipid metabolism	Atherosclerosis [178]
Lunasin	EA.hy926 cells and apolipopro-tein E deficiency (*ApoE*^−/−^) male mice fed a HFD	Upregulated HO-1 via the PI3K/AKT1/NRF2/ARE pathway, attenuating H_2_O_2_ and apoptosis	Atherosclerosis [130]
Metformin	Male C57BL/6 J mice	Ameliorated obesity phenotype and metabolic disorders, reduced the heart weight index, and attenuated cardiac fibrosis	Obesity [179]
Momordicine I	Neonatal rat cardiac fibroblasts	Abolished fibroblast proliferation and collagen synthesis	Hyperglycemia [180]
Parthenolide	3T3-L1 and RAW264.7 cells. Male C57BL/6 J mice	In cells, suppressed inflammatory responses by downregulating IL6 and CCL2. In animals, reduced body weight and WAT, downregulating NF-κB and MAPKs	Obesity [69]
Pentamethylquercetin	CD1 male and female mice treated with monosodium glutamate	Ameliorated obesity phenotypes, decreased the heart wall thickness, and attenuated cardiac fibrosis	Obesity [181]
Pterostilbene	HUAEC and Male Sprague-Dawley rats with endothelial injury of the iliac arteries and feeding with a 2.5% cholesterol diet with 1% glucose	In vitro, decreased oxidative stress injury and apoptosis. In animals, decreased inflammation, atherogenesis, reduced aortic plaque size, reduced macrophage infiltration, and suppressed oxidative stress and apoptosis	Atherosclerosis [182]
Spiraeoside	AC16 cells	Inhibited ROS and MDA production, increased activities of SOD1, GPX1, and CAT. Prevented apoptosis	Hyperglycemia [183]
Tanshinone IIA	Neonatal rat cardiac fibroblasts	Abolished cell proliferation and collagen synthesis via activation of NRF2 and inhibition of TGFB1 production and SMAD2/3 phosphorylation	Hyperglycemia [184]
Z-Ligustilide	EA.hy926 cells and HFD-fed *Ldlr*-deficient male mice	In vitro, alleviated oxidative stress and cell injury caused by t-BHP. In vivo, restrained atherosclerosis progression, attenuated atherosclerotic plaque formation, alleviated lipid peroxidation, and increased antioxidant enzyme activity in aortas	Atherosclerosis [185]

Abbreviations: AKT1, AKT serine/threonine kinase 1, also known as protein kinase B (PKB); PRKAA2, AMP activated protein kinase; ANG II, angiotensin II; ARE, antioxidant response element; BACH1, BTB domain and CNC homolog 1; eNOS, endothelial nitric oxide synthase; ER, endoplasmic reticulum; HUVECs, human umbilical vein end endothelial cells; HUAEC, human umbilical artery endothelial cells; HFD, high-fat diet; MDA, malondialdehyde; MAPKs, mitogen-activated protein kinases; PPARG; PPARG coactivator 1 alpha; PI3K, phosphatidylinositol 3-kinase; ROS, reactive oxygen species; SHRs, spontaneously hypertensive rats; STZ, streptozotocin; t-BHP, tert-butyl hydroperoxide; TGFB1, transforming growth factor beta-1; WAT, white adipocyte tissue; WKY, Wistar Kyoto.
